# Supplementary material for: Influence of Single Dose Enrofloxacin Injection on Development of Fluoroquinolone Resistance in Campylobacter jejuni in Calves
Source: Antibiotics (Basel). 2022 Oct 13;11(10):1407. doi: 10.3390/antibiotics11101407 (PMC9598087; doi:10.3390/antibiotics11101407)
Supplement: Supplementary file 1 [file antibiotics-11-01407-s001.zip › antibiotics-1943849-supplementary.pdf]

Table S1. Bacterial isolates used for inoculation of calves with *Campylobacter jejuni* (oral) and *Mannheimia haemolytica* (trans-tracheal) in the current study.

Isolate/strain	Source	Origin	Isolation date	Reference
C. jejuni IA-6-FC-30	Feces of healthy cattle	Iowa	2013	[61]
C. jejuni MO-2-FC-25	Feces of healthy cattle	Missouri	2013	[61]
M. haemolytica VDL4004	Pneumonic lung of calf	Iowa	2018	[44]
